# Microglia and Macrophages in Malignant Gliomas: Recent Discoveries and Implications for Promising Therapies

**DOI:** 10.1155/2013/264124

**Published:** 2013-06-25

**Authors:** Anna Carolina Carvalho da Fonseca, Behnam Badie

**Affiliations:** ^1^Laboratório de Morfogênese Celular, Instituto de Ciências Biomédicas, Universidade Federal do Rio de Janeiro, 21941-902, Rio de Janeiro, RJ, Brazil; ^2^Division of Neurosurgery and Department of Cancer Immunotherapeutics & Tumor Immunology, City of Hope Beckman Research Institute, Duarte, CA 91010, USA

## Abstract

Malignant gliomas are the most common primary brain tumors. Their deadliest manifestation, glioblastoma multiforme (GBM), accounts for 15% of all primary brain tumors and is associated with a median survival of only 15 months even after multimodal therapy. There is substantial presence of microglia and macrophages within and surrounding brain tumors. These immune cells acquire an alternatively activated phenotype with potent tumor-tropic functions that contribute to glioma growth and invasion. In this review, we briefly summarize recent data that has been reported on the interaction of microglia/macrophages with brain tumors and discuss potential application of these findings to the development of future antiglioma therapies.

## 1. Introduction

Malignant gliomas, the most common primary brain tumors that arise from glial cells within the central nervous system (CNS), are among the most fatal human cancers [1]. Glioblastoma multiforme (GBM), the most aggressive type of malignant glioma, is highly invasive, making tumor recurrence certain even after a complete resection [2]. Besides, the presence of the blood-brain barrier (BBB) significantly limits the penetration of most chemotherapeutic agents into the CNS [3]. With a median survival of only 14.6 months even after aggressive therapy with surgery, radiation, and chemotherapy, most patients succumb to their disease within two years of the initial diagnosis [4]. Thus, there is a pressing need for discovery of more effective therapies to improve patient outcomes.

Malignant gliomas are heavily infiltrated by myeloid-derived cells (recently reviewed by Kushchayev et al. [5]). Among these, tumor microglia and macrophages appear to be the most common cells in brain tumors. Tumor microglia arise from resident CNS macrophages, while circulating monocytes give rise to glioma-associated macrophages. In experimental glioma models, tumor microglia and macrophages can be differentiated by FACS based on CD45 and CD11b staining characteristics [6], but in human tissue samples, such separation is not as distinct. Although both cell types can acquire M1 phenotype and are capable of releasing proinflammatory cytokines, phagocytosis, and antigen presentation [7], their effector immune function in gliomas appears to be suppressed. In fact, increasing new evidence suggests that microglia and macrophages interact with the tumor cells by promoting their growth and migration [8]. In this review, we briefly summarize recent data that has been reported on microglia/macrophages brain tumor interaction and discuss potential application of these findings to the development of future antiglioma therapies. 

## 2. Chemoattraction

 Glioma-associated microglia and macrophage (collectively referred to as GAMs here) compose approximately 30% of tumor inflammatory cells and are actively recruited by gliomas through secretion of a variety of factors including chemokines, cytokines, and matrix proteins [9–13]. Among chemokine pathways involved in TAM chemoattraction, CCL2 (monocyte chemotactic protein-1 (MCP-1)) was among the first identified in gliomas [14]. Although CCL2 expression can be induced by a variety of stimuli and cytokines, mechanisms responsible for its baseline expression by gliomas are being studied. Adenosine-5′-triphosphate (ATP), for example, was shown to stimulate the production of chemokines MCP-1 and interleukin-8 (IL-8) in gliomas [15]. Recently, we demonstrated that in a subgroup of gliomas, protein S100 calcium binding protein B (S100B) may also play a role in MCP-1 upregulation and GAM recruitment [16]. A direct correlation between the percentage of GAMs and MCP-3 expression levels has also been demonstrated in human gliomas, suggesting MCP-3 to also participate in microglia/macrophages chemoattraction [12].

 Stromal-derived (SDF-1) factor-1 is another chemokine that has been shown to promote microglia/macrophage trafficking in gliomas [17]. Trying to recapitulate neuropathological features of human high-grade glioma, Wang et al. established a new murine brain tumor model, ALTS1C1, which expresses high levels of SDF-1. To unveil the role of SDF-1 in this tumor model, the expression of this chemokine in tumor cells was inhibited. The density of microglia/macrophages in the SDF-knockdown tumor was higher in nonhypoxic than in hypoxic regions, suggesting that SDF-1 production by tumor cells might be crucial for the accumulation of microglia/macrophages into areas of hypoxia and tumor invasiveness [13].

 Glioma and GAMs participate in a number of paracrine networks that promote their coexistence. Glioma cells constitutively express colony stimulating factor-1 (CSF-1) that stimulates microglia invasion through its receptor CSF-1R. Synergistically, microglia stimulate glioma cell invasion through epidermal growth factor receptor (EGFR) activation [10]. Further, in response to glioma cells, microglia express tumor necrosis factor receptor of mouse embryo (TROY) that drives microglia migration towards glioma cells [18]. Also, the chemokine CX3CL1 expressed in glioblastoma cells promotes recruitment of human microglia/macrophages through its receptor CX3CR1 and enhances the expression of matrix metalloproteases 2, 9, and 14 in these cells, possibly promoting tumor invasion [11].

 Glioma-initiating and cancer stem cells also have a role in recruiting microglia/macrophages. The former promote microglia migration through chemokines CCL5, vascular endothelial growth factor (VEGF) and neurotensin (NTS) release [19], while conditioned medium from the latter was shown to induce the migration of human monocytes [20]. 

## 3. Immunosuppression

 After attracting microglia/macrophages, tumor cells establish an immunosuppressed microenvironment, leading GAMs to acquire an alternatively activated (M2) phenotype that further contributes to the local immunosuppression and supports tumor growth and invasion [8, 21, 22]. Recently, we demonstrated that S100B, a protein that is expressed by most gliomas and activates receptor for advanced glycation end products (RAGE) on microglia/macrophages, can induce signal transducer and activator of transcription 3 (STAT3) activity, resulting in suppression of microglia and primary monocyte function *in vitro*, reflected by inhibition of interleukin-1 beta (IL-1*β*), tumor necrosis factor-alpha (TNF-*α*) production, and other pro-inflammatory cytokines [23]. As a signal transducer, STAT3 is a central node for numerous oncogenic signaling pathways involving cytokines and growth factors [24]. STAT3 is also an important transcription regulator, defining a transcriptional program at multiple levels that facilitate tumor cell proliferation, survival, invasion, cancer-promoting inflammation, and suppression of antitumor immune responses [24]. 

GAMs have also been shown to express a variety of immunosuppressive cytokines. For example, transforming growth factor-beta 1 (TGF-*β*1), an immunosuppressive cytokine that is expressed by glioma cells, is also produced by GAMs [25]. This process may be in part mediated by release of macrophage inhibitory cytokine-1 (MIC-1) by glioma stem cells [20]. 

## 4. Promotion of Tumor Growth and Invasion

 Wesolowska et al. have shown that TGF-*β*1 originated from microglia is crucial for promotion of glioma invasion. Interestingly, primary microglia cells cocultured with glioma cells drastically increased the secretion of TGF-*β*1 [26]. More recently, another research group confirmed that the TGF-*β*1 released by microglia/macrophages enhances the invasive capacity of the CD133^+^ glioma cells (glioma stem-like cells) more than the CD133^−^ committed cells, and that this process most likely occurred through type II TGF-*β* receptor. Moreover, this invasion was suggested to be promoted by an increase of MMP-9 (matrix metalloprotease 9) expression in the CD133^+^ glioma cells [27].

Metalloproteases play an important role in microglia/macrophage-mediated glioma migration. Conditioned medium from microglia cells incubated with S-Adenosylhomocysteine (SAH) promotes the invasion of a glioma cell line in a SAH-dose-dependent manner. This occurs through the increase of MMP-2 expression and activity and the decrease of the tissue inhibitor of metalloprotease-2 (TIMP-2) in microglia-treated cells [28]. Markovic and colleagues have elegantly shown that membrane type I metalloprotease (MT1-MMP) is upregulated in GAMs and that the tumor itself induces the expression and activity of MT1-MMP [29]. This microglial metalloprotease in turn seems to activate MMP-2 in gliomas, leading to even higher tumor invasiveness [29]. GAMs also express CX3CR1 and, in response to CX3CL1, increase their adhesion, migration, and expression of MMP-2, -9, and -14 [11].

 Cytokines and chemokines are also important in glioma growth and invasion. Overexpression of CCL2 in the U87 glioma cell line was recently shown to enhance its invasiveness in a three-dimensional collagen matrix when these cells were cocultured with microglia (which express the CCL2 receptor CCR2) [30]. Furthermore, levels of IL-6 were increased in the coculture medium, and the expression of IL-6 *in situ* corresponded to the microglia/macrophages. Also, incubation of the glioma cells with recombinant IL-6 significantly increased their invasion, a process that was reversed with an IL-6 neutralizing antibody. These findings suggest that gliomas exploit microglia/macrophages through a CCL2/CCR2/IL-6 loop to increase their invasion and migration [30]. Intense angiogenesis is another hallmark of malignant gliomas whereby these tumors obtain essential nutrients and oxygen [31]. Loss of Flt-1, a VEGF receptor, signaling in microglia/macrophages leads to a decrease in tumor growth and vessel density, confirming an active role of microglia/macrophages in glioma angiogenesis [32].

 Recently, it was demonstrated that the cochaperone stress inducible protein-1 (STI1), a cellular prion protein (PrP^c^) ligand, released by primary microglia cells promotes proliferation and migration of glioma cell lines in a PrP^c^-independent fashion (possibly involving MMP-9). Moreover, this proliferative effect was specific to brain macrophages, since conditioned medium from peritoneal macrophages was unable to induce glioma cells growth [33]. As previously discussed, glioma and microglia interactions mediated through EGF and CSF-1 can also increase tumor invasion [10]. 

## 5. Promising New Therapies

 As detailed in this and other reviews, microglia/macrophages are recruited to the glioma site, but their immune effector function is impaired, and these cells can even promote tumor growth and invasion. Attempts are being made to identify new targets that can reverse this GAM function. One potential candidate is STAT3. Our group showed that conditioned medium from glioma cells increased STAT3 activity in microglia cells, resulting in overexpression of IL-6 and IL-10 and downregulation of IL-1*β* [34]. When STAT3 was inhibited *in vitro* by a pharmacological agent or small interfering RNA (siRNA), the expression profile of these cytokines was reversed. Moreover, silencing of STAT3 in a murine glioma model resulted in microglia/macrophages activation and tumor growth inhibition [34]. Likewise, corosolic and oleanolic acids have been shown to suppress STAT3 in macrophages [35, 36]. These compounds also inhibited the expression of CD163, a marker of the M2 phenotype and the secretion of IL-10 in human macrophages, suggesting that they could potentially be used to suppress the M2 polarization of microglia/macrophages [35, 36]. 

 The use of antibodies to alter GAM function also has been under investigation. Systemic administration of neutralizing antibody against CCL2 significantly inhibits the infiltration of microglia/macrophages in mice bearing gliomas [37]. Furthermore, the combination of anti-CCL2 therapy with chemotherapy (temozolomide) significantly prolonged the survival of mice, suggesting a possible new therapeutic approach [37]. Furthermore, antiphosphatidylserine antibody combined with irradiation was also investigated in a rat model of glioblastoma. Antiphosphatidylserine binds to tumor vascular cells exposing phosphatidylserine in response to irradiation and induces antibody-dependent cell-mediated cytotoxicity by CD11b-positive macrophages. This resulted in the death of tumor cells through starvation and significantly increased the median survival time of tumor-bearing animals [38].

 Taking advantage of their phagocytic properties, our group is actively pursuing the development of nanoparticles for modulation of microglia/macrophage activity in brain tumors. Carbon nanotubes (CNTs) and cyclodextrin-based polymer are semiselectively taken up by GAMs with no toxicity [39, 40]. These nanoparticles can be used to enhance the uptake of CpG oligonucleotides, an agonist of toll-like receptor 9 (TLR-9), by glioma-associated inflammatory cells. This strategy was used to overcome the local tumor immunosuppression and eradicated gliomas in mice models [41].

Moreover, Kopatz et al. used another approach to boost the phagocytosis property of microglia cells in a glioma model. Microglia were stimulated to express sialic-acid-binding immunoglobulin-like lectin-h (Siglec-h) that bound to intact glioma cells, but not normal brain cells, resulting in the engulfment of tumor cells, a process dependent on DAP12, an adapter molecule, signaling [42].

 Microglia/macrophages may also function as delivery vehicle into brain tumors [43]. Baek and colleagues reported using murine macrophages as carriers of nanoshells (NS) into gliomas for photothermal ablation. NS composition efficiently converts absorbed near-infrared light (NIR) into heat and exposure of glioma spheroids infiltrated with NS-loaded macrophages to NIR laser resulted in complete tumor growth inhibition [44].

 Other well-known agents can also be used as antiglioma therapies by targeting GAMs. The antibiotic minocycline hydrochloride is a small, highly lipophilic molecule that is readily absorbed after oral administration and capable of crossing the BBB. Markovic et al. have shown that minocycline attenuates the protumorigenic effect of GAMs by inhibiting p38 MAP kinase that is responsible for MT1-MMP upregulation in microglia [45]. Besides, the inhibition of p38 MAPK was also shown to reduce the secretion of pro-inflammatory cytokines from microglia and tumor cells, resulting in a decrease of glioma migration [46]. Moreover, systemic administration of cyclosporine A (CsA) was shown to decrease glioma growth and angiogenesis by inhibiting microglia/macrophages infiltration by inducing cell death and blocking the expression and activity of important enzymes and cytokines required for tumor invasion [47]. Propentofylline (PPF), an atypical methylxanthine and glial modulating agent with anti-inflammatory actions, was also shown to be effective in reducing glioma growth by targeting microglia and possibly decreasing their expression of MMP-9 [48]. 

 Other immunotherapy approaches that target GAMs include stimulation of microglia by IL-12 to increase their phagocytic activity and TNF-related apoptosis inducing ligand (TRAIL) release [49]. Also, stimulation of human GAMs by the TLR3 agonist poly(I:C) results in tumor cell death and inhibition of tumor cell growth and invasion [50]. Finally, because microglia/macrophages express folate receptor *β* (FR*β*), a recombinant immunotoxin to FR*β* has been used to deplete GAMs in order to decrease tumor growth [51]. 

Integrins can also be targeted to attenuate glioma growth. In an organotypic brain culture containing glioma cells, the inhibition of alpha5 beta1 (*α*5*β*1) integrin resulted in a significant decrease in tumor growth. Interestingly, depletion of microglia cells abolished the effect of the *α*5*β*1 inhibitor on glioma invasion, suggesting that the *α*5*β*1 integrin promotes tumor development through interactions with microglia [52].

 As new novel strategies are being developed to target GAMs, recent reports suggest the existence of a therapeutic window when such approaches can be utilized. It seems that microglia/macrophage functional impairment may occur relatively late in the course of glioma growth, suggesting that early intervention to target these cells may provide the best therapeutic opportunity [53, 54]. 

## 6. Conclusion

GAMs appear to be a heterogeneous cell population with diverse roles in gliomatogenesis. These cells are actively recruited by gliomas and participate in tumor growth, invasion, angiogenesis, and local immunosuppression. A better understanding of their function will be helpful in developing novel therapies against malignant gliomas. 
